# Application of Spectral Domain Optical Coherence Tomography to Objectively Evaluate Posterior Capsular Opacity In Vivo

**DOI:** 10.1155/2018/5461784

**Published:** 2018-12-23

**Authors:** Shasha Yu, Chengzhe Lu, Xin Tang, Xiaoyong Yuan, Bo Yuan, Zhe Yu

**Affiliations:** ^1^Clinical College of Ophthalmology, Tianjin Medical University, Tianjin 300020, China; ^2^Tianjin Eye Hospital, Tianjin 300020, China; ^3^Tianjin Key Laboratory of Ophthalmology and Visual Science, Tianjin, China; ^4^Tianjin Eye Institute, Tianjin, China

## Abstract

**Objectives:**

To objectively evaluate posterior capsular opacification (PCO) with RTVue-100 spectral domain-OCT and assess the agreement with the Pentacam system.

**Methods:**

Sixty-seven eyes diagnosed with PCO were included. RTVue-100 SD-OCT was used to scan the IOL outline and PCO at horizontal and vertical meridians. PCO was also imaged with a Pentacam and slit-lamp photography system. With RTVue-100 SD-OCT, the PCO area, thickness, density, and objective scores were recorded and used to evaluate the severity of PCO at 3 mm and 5 mm diameter ranges of the IOL optic region. We assessed the correlation of visual acuity, PCO characteristics, and PCO scores. PCO scores acquired from RTVue-100 SD-OCT images were also compared with those from the Pentacam. Differences between pear-type and fibrosis-type PCOs were also compared using RTVue-100 SD-OCT cross-sectional images.

**Results:**

The cross-sectional images of PCO acquired with RTVue-100 SD-OCT corresponded well to Pentacam and slit-lamp retroillumination images. IOL-posterior capsular space, area, thickness, and density of the proliferated and accumulated LECs could be clearly visualized and quantified with RTVue-100 SD-OCT. PCO scores were correlated with decreased visual acuity, which was in line with the outcomes using the Pentacam. Differences between the pear-type and fibrosis-type PCO were statistically significant; pear-type PCOs showed a wider and thicker opacification region with lower density compared with fibrosis-type PCOs.

**Conclusion:**

RTVue-100 SD-OCT could be a powerful tool in PCO objective evaluation and classification. OCT could be used to visualize the morphology and outline of PCO. Thus, it could discriminate and quantify differences between different types of PCO. PCO scores seem to be a useful factor that could reliably reflect PCO severity.

## 1. Introduction

Posterior capsular opacification (PCO) is one of the most common complications after cataract surgery, which can be treated with neodymium-yttrium-aluminium-garnet (Nd:YAG) laser capsulotomy. However, the increased cost of treatment, risk of retinal detachment [1], and macula oedema occurrence cannot be ignored [2]. Enormous efforts have been focused on exploring methods to prevent the formation of PCO [3, 4]. Theoretically, PCO is the result of migration and proliferation of LECs. The severity should include opacification area, thickness, and density. However, until now, the extent of posterior capsular opacification is mostly subjectively or semiquantitatively evaluated [5, 6], and only the area or objective density is used. Thus, an objective method to evaluate PCO is still needed [7, 8].

OCT is a noninvasive technique based on low-coherence interferometry that could generate high-resolution cross-sectional images from backscattered light, thus benefiting ophthalmologists in evaluating different retinal diseases and assessing changes in the anterior segment of the eye [9, 10]. It was reported that OCT1 might be useful for quantifying PCO [11]. The in vitro ultrahigh-resolution optical coherence tomography (UHR OCT) with a wavelength of 800 nm was suggested to be meaningful when evaluating the pseudophakic human eye, lens capsule, and intraocular lens (IOL). By using OCT, the severity of PCO and the extent of IOL apposition to the posterior capsule could also be visualized [12].

However, PCO quantification was necessary and meaningful. RTVue-100 SD-OCT could provide detailed information on the cloudy posterior capsule with cross-sectional images, which allows precise evaluation of the opacification area, thickness, and density. Even the area of IOL-posterior capsular space could be quantified.

We therefore aimed to assess the application of RTVue-100 OCT in a PCO severity quantification study and compare the images among RTVue-100 OCT, Pentacam, and slit-lamp methods in PCO evaluation and classification.

## 2. Materials and Methods

### 2.1. Patients

This prospective observational study recruited 67 patients (67 eyes) implanted with a posterior capsular IOL who had been diagnosed with PCO and planned to receive Nd:YAG capsulotomy surgery. The study was approved by the Research Review Board and strictly followed the tenets of the Declaration of Helsinki (1989) of the World Medical Association. Informed consent was collected before Nd:YAG capsulotomy surgery. A total of 10 eyes of 10 patients received uneventful phacoemulsification and IOL implant surgery without PCO and were enrolled as controls.

### 2.2. Patient Examination

Routine examinations were done by an expert ophthalmologist. Exclusion criteria were as follows: cornea opacity, uveitis, glaucoma, eventful cataract surgery, eye with poor fixation ability, a history of eye trauma and complicated surgery, and a history of severe systemic disease. Visual acuity (VA) was recorded using a Snellen E chart before mydriasis and was converted to the logarithm of the minimum angle of resolution (logMAR) value for statistical analysis. Retroillumination photography, Pentacam images, and RTVue-100 OCT images were acquired after pupil dilation.

### 2.3. Retroillumination with Slit-Lamp Examination

Retroillumination photographs of the anterior segment, especially the posterior capsular region, were obtained with the slit-lamp photograph system (BX900, HAAG-STREIT company, Switzerland) after pupil dilation.

### 2.4. Pentacam Examination

**Figure 1:**
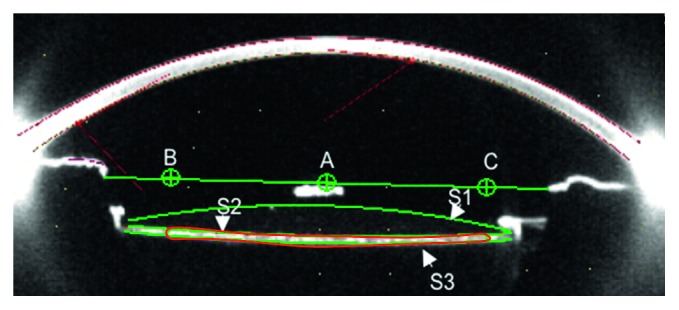
Illustration graph of the PCO evaluation method with the Pentacam (the figure was acquired at the horizontal meridian). Measurement of PCO characteristics in one cross-sectional image. S: surface; S1: anterior surface of IOL; S2: posterior surface of IOL; S3: posterior capsule. Point A is the centre of the horizontal line, which is the centre of the pupil, and the distance between points B and C is 5 mm of the central optic region. The PCO area is located between S2 and S3, which is indicated with a red line.

A Pentacam system (Pentacam, Oculus, Germany) was used in combination with the Scheimpflug video-photography system and a monochromatic slit-light source that provided a 3-dimensional scan of the anterior segment of the eye. In brief, the Scheimpflug slit images of the anterior segment were taken with a rotating camera from 0 to 360 degree meridians after pupil dilation. Twenty-five images were taken in 2 seconds, and eye movement was automatically corrected during imaging processing. Patients were told to not blink their eyes during measurement. After measurement, the high-quality images at the horizontal and vertical meridians were selected and transferred to a computer for further analysis. The area and density of PCO at a diameter of 5 mm within the IOL optic region were evaluated using Image Pro Plus software 6.0. The opacification density values were expressed as computer compatible tape, and the scattered light intensity ranged from 0 to 255 [13]. PCO density assessment was as follows. To eliminate the scattered light intensity influence of the IOL itself, the average light scatter density of the central 5 mm ∗ 0.25 mm area of the IOL was first evaluated and then subtracted from the average PCO density at 5 mm of the IOL optic region. The PCO area and density values at the two meridians were then averaged for comparison (details in Figure 1).

PCO score definition: PCO score = PCO area × (PCO density−scatter light intensity)

### 2.5. RTVue-100 OCT Examination

**Figure 2:**
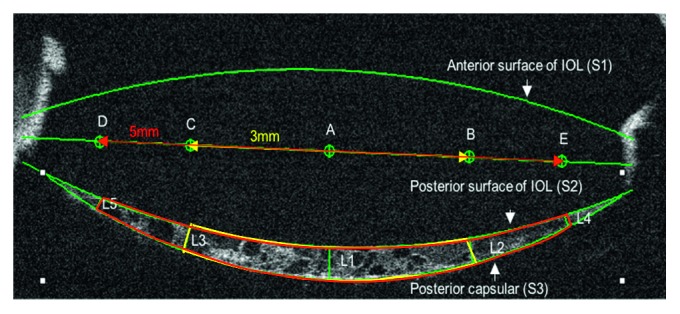
Illustration graph of the PCO evaluation method RTVue-100 OCT (the figure was acquired at the horizontal meridian from the same eye as in Figure 1). Measurement of PCO characteristics in one cross-sectional image. S: surface. Point A is the centre of the line (green line) that passed the horizontal or vertical centre of the IOL, which is the centre of the IOL optic region. Distance between points B and C is defined as the 3 mm diameter range of the IOL optic region (yellow arrow line), while the red line between points D and E is the 5 mm range (red arrow line). S1 is the anterior surface of the IOL, S2 is the posterior surface of the IOL, and S3 is the posterior capsule. The crescent-shaped space between S3 and S2 is the IOL-posterior capsular space, which is the space over the full 6 mm diameter. The lengths of L1, L2, L3, L4, and L5 are PCO thicknesses at the point of the central optic region and at the 3 mm and 5 mm diameter of the IOL optic region. The area between L3, L2, S2, and S1 is the PCO region at the 3 mm diameter range of the IOL optic region (indicated with a yellow line), while the area between L4, L5, S2, and S3 is the PCO region at the 5 mm diameter range of the IOL optic region (indicated with a red line).

RTVue-100 OCT (RTVue-100, Optovue Inc, Fremont, CA), based on the spectral domain technique with a corneal anterior module long adaptor lens (1.96 mm scan depth and 6 mm scan width), was adopted in our study. A super luminescence diode emitted light with a 50 nm bandwidth was centred at 830 nm and had a scan speed of 26,000 axial scans per second with an axial resolution of 5 *μ*m. A corneal anterior module long adaptor lens was mounted on the probe and focused on the anterior segment part, including the IOL and PCO after pupil dilation. Patients were told to fixate straight ahead on the red light using the fellow eye [14]. Using a cornea cross line mode, images of the IOL and PCO on the vertical and horizontal meridians crossing the central cornea were taken, and then images were transferred to a personal computer for analysis using Image Pro Plus software 6.0. The area between the IOL and posterior capsular region was evaluated. PCO area and density at the 3 mm and 5 mm diameter ranges of the IOL optic regions were measured. PCO thickness at the 3 mm and 5 mm point of the IOL optic regions were measured. The area, thickness, and density values of the two meridians were averaged for further comparisons (details in Figure 2). The PCO score definition was the same as that of the Pentacam system.

### 2.6. Statistical Analysis

Statistical analyses were performed with IBM SPSS Statistics 23.0. The Kolmogorov–Smirnov test was used to assess the distribution of all data. A nonparametric Spearman test was used to evaluate the relationship among visual acuity, PCO characteristics (area, thickness, and density), and IOL-posterior capsular spaces measured with RTVue-100 OCT, as well as the relationship between PCO characteristics measured with RTVue-100 OCT and the Pentacam. A Mann–Whitney *U* test was used to compare differences between pear-like PCO and fibrosis-type PCO. *p* values less than 0.05 were considered significant.

## 3. Results

The study analysed PCO characteristics of 67 pseudophakic eyes measured with RTVue-100 OCT. The average age of the patients was 66.91 ± 11.10 (ranging from 37 to 91) years old. The average PCO formation time was 35.79 ± 24.15 months. The average visual acuity was 0.40 ± 0.11 (ranging from 0.15 to 0.52).

**Figure 3:**
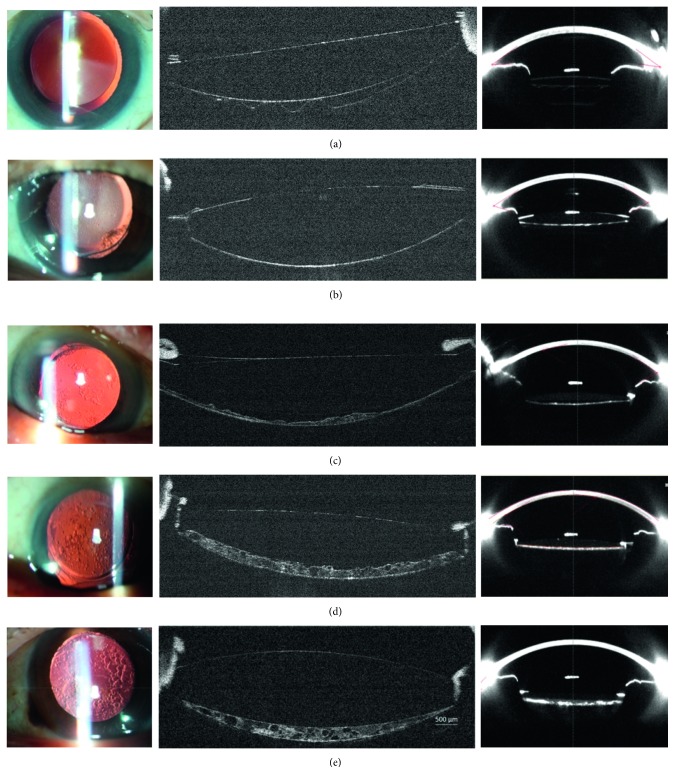
PCO images measured with different methods. The first column shows retroillumination images, the second demonstrates RTVue-100 OCT images, and the third corresponds to Pentacam images; (a) pseudophakic eye without PCO; (b) fibrosis-like; (c) pear-like, mild; (d) pear-like, middle; (e) pear-like, severe.

PCO could be clearly visualized and classified by morphology with images measured by RTVue-100 OCT (Figure 3, second column), which was parallel with the retroillumination photograph system (Figure 3, first column) and the Pentacam (Figure 3, third column). PCO measured with RTVue-100 OCT could be described as follows: fibrosis PCO with enhanced intensity of the posterior capsule in 14 patients. Pear-like PCO with accumulation of proliferated LEC, extracellular matrix, and bladder cells in the IOL-posterior capsular space occurred in 46 patients, and 7 patients had the mixture type (for further analysis, the mixture type was excluded).

**Figure 4:**
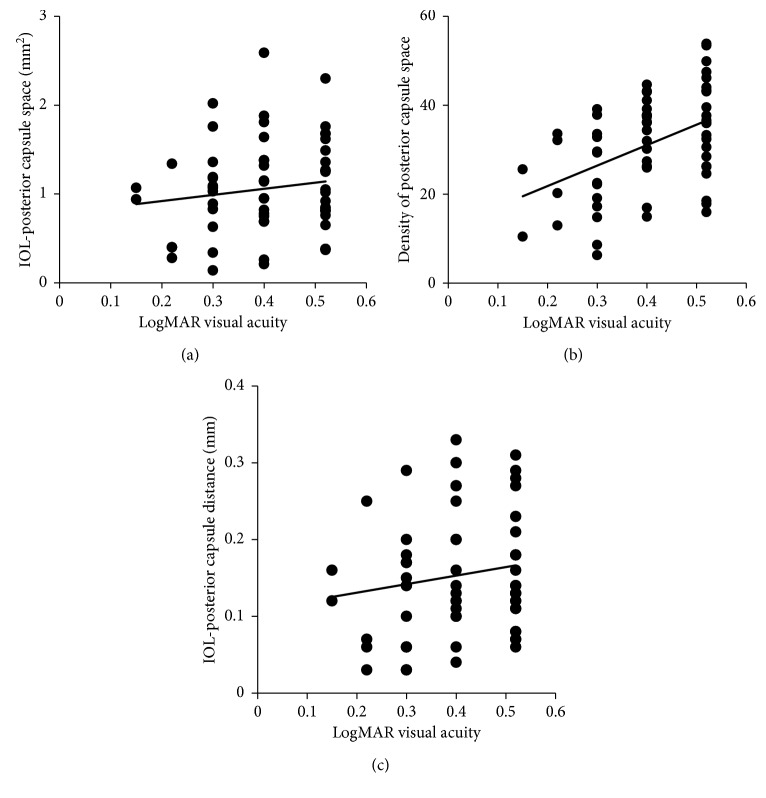
Correlation analysis between visual acuity and IOL-posterior capsule space. (a) Correlation between visual acuity and the area of the IOL-posterior capsule space using RTVue-100 OCT. Spearman's correlation analysis reported that the correlation coefficient was 0.11 (*p*=0.42). (b) Correlation between visual acuity and the density of the IOL-posterior capsular space. The correlation coefficient was 0.42 (*p* ≤ 0.001). (c) Correlation between visual acuity and the IOL-posterior capsular distance. The correlation coefficient was 0.14 (*p*=0.29).

PCO characteristics measured with RTVue-100 OCT are shown in Table 1 and 2. The average IOL-posterior capsular distance was 0.15 ± 0.08, ranging from 0.03 to 0.33 mm. The mean area of the IOL-posterior capsular space was 1.06 ± 0.53, ranging from 0.14 to 2.59 mm^2^. The density of the IOL-posterior capsular space was 32.05 ± 14.12 (6.26 to 99.78), which was significantly correlated with decreased visual acuity (*r*=0.42, *p*=0.001), as described in Figure 4(b).

**Figure 5:**
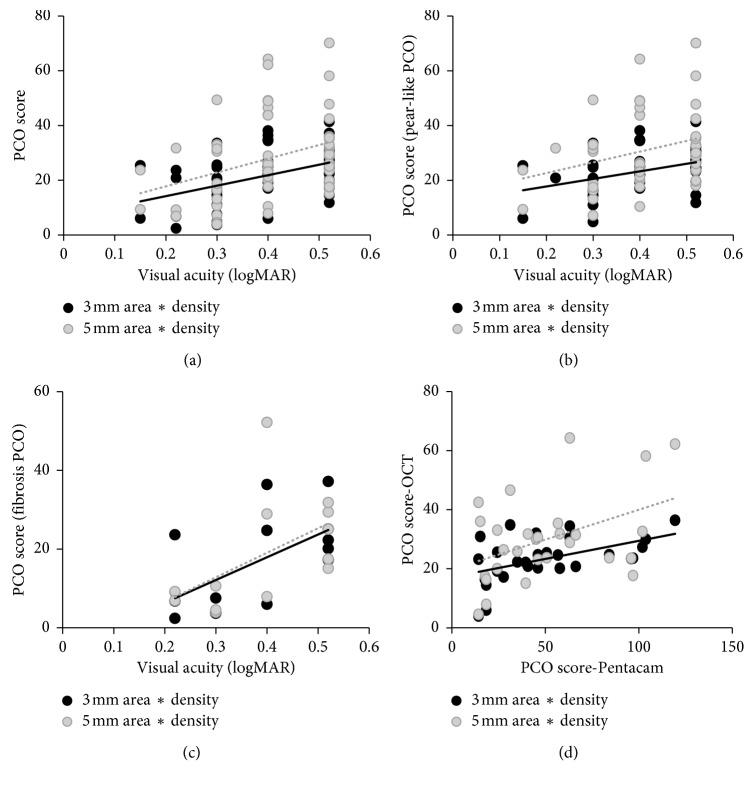
Relationship between visual acuity and PCO scores at the 3 mm and 5 mm IOL optic regions. Spearman's correlation analysis was used. (a) Visual acuity and PCO scores for all PCO (*r*=0.43, *p* ≤ 0.001; *r*=0.38, *p* ≤ 0.001); (b) visual acuity and PCO scores for pear-type PCOs (*r*=0.36, *p*=0.01; *r*=0.29, *p*=0.05); (c) visual acuity and PCO scores for fibrosis-type PCOs (*r*=0.56, *p*=0.04; *r*=0.67, *p*=0.01); (d) PCO scores with OCT and PCO scores with the Pentacam (*r*=0.41, *p*=0.03; *r*=0.22, *p*=0.24). PCO score = PCO area × (PCO density−light scatter intensity).

We defined the PCO score by multiplying the PCO area by the mean density. Spearman's correlation analysis showed that visual acuity was correlated with PCO scores both at the 3 mm (*r*=0.43, *p* ≤ 0.001) and 5 mm (*r*=0.38, *p* ≤ 0.001) diameters of the IOL optic region. For pear-like cases, the correlation efficient of PCO score was 0.36 (*p*=0.01) and the fibrosis PCO was 0.56 (*p*=0.04) at the 3 mm diameter of the IOL optic region (Figure 5). In addition, the decreased visual acuity was correlated with the PCO area, density, and thickness, although the correlation efficient was lower.

Comparisons between Pentacam and RTVue-100 OCT for PCO severity evaluation: Spearman's correlation analysis showed that PCO scores from the Pentacam (3 mm IOL optic region) were correlated with those from RTVue-100 OCT both at the 3 mm and 5 mm (*r*=0.40, *p*=0.03; *r*=0.21, *p*=0.28) diameters of the IOL optic regions (Figure 5(d)).

**Figure 6:**
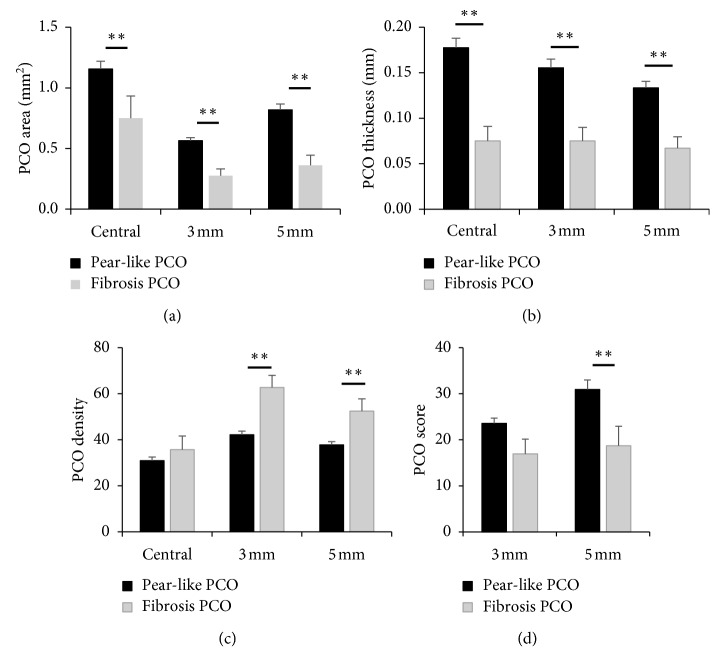
Comparison between pear-type and fibrosis-type PCO. (a) Area of the two types of PCO; central refers to the full area of the IOL-posterior capsular space. 3 mm and 5 mm refer to the area of the 3 mm and 5 mm diameter range of the IOL optic region. (b) Thickness of the two types of PCO; central refers to the central point of the full IOL optic region and 3 mm and 5 mm refer to the point at the 3 mm and 5 mm diameter range of the IOL optic region. (c) Density of the two types of PCO; central refers to the full area of the IOL-posterior capsular space. 3 mm and 5 mm refer to the area of the 3 mm and 5 mm diameter range of the IOL optic region. (d) Objective score of the two types of PCO. A Mann–Whitney *U* test was used to compare the differences. ^*∗∗*^*p* < 0.01.

In comparisons between fibrosis PCO and pear-like PCO, the central distance and full area of the IOL-posterior capsular spaces were significantly different (*z*=−4.69, *p* ≤ 0.001; *z*=−2.88, *p*=0.004). The PCO area, thickness, and density at 3 mm (*z*=−4.07, *p* ≤ 0.001; *z*=−3.82, *p* ≤ 0.001; *z*=−4.14, *p* ≤ 0.001) of the IOL optic region were significantly different, as well as the PCO at the 5 mm IOL optic region. PCO scores were 23.57 ± 7.78 for the pear-like type and 16.94 + 11.99 for the fibrosis type, and these differences were not statistically significant at the 3 mm IOL optic region (*z*=−1.89; *p*=0.06). Details of comparisons between fibrosis PCO and pear-like PCO are described in Figure 6.

## 4. Discussion

OCT has been used to diagnose posterior polar cataracts [15], evaluating IOL tilt and decentration [16], assessing capsular bend formation [17, 18], and even evaluating PCO severity [19–21]. In our study, RTVue-100 OCT with an improved axial resolution of 5 *μ*m and a scan speed of 26,000 axial scans per second provided an accurate objective assessment of PCO. We found that, in RTVue-100 OCT acquired images, the IOL outline, opacified posterior capsule, the accumulated extracellular substance, and LECs could be clearly visualized. Meanwhile, the severity of PCO could also be quantified. In addition, these images were well in line with those from the retroillumination system and the Pentacam.

Based on RTVue-100 OCT images, we first evaluated the central distance, area, and density of the IOL-posterior capsular space, which were correlated with decreased visual acuity. The existence and influence of this space on visual function are in agreement with the concept “no space, no cell, no PCO” [22]. Studies [23, 24] proved that a more adhesive material could promote the elimination of IOL-posterior capsular space and prevent LEC migration and proliferation. In our study, the residual IOL-posterior capsular space and PCO may have been due to the poor or delayed adhesion of the IOL to the capsular space [18].

When evaluating PCO severity, we found that PCO density was a significant factor that correlated with decreased visual acuity. Studies have reported that, with increasing severity of PCO, visual acuity and stray light deteriorate [25]. Hayashi's study [26] proved good correlations between visual acuity and PCO density with the EAS-1000 method. However, it was also reported that visual acuity was significantly correlated with posterior capsular thickening (PCT), but not PCO density [11]. The results were different from ours. This difference may be explained by different evaluation methods. With OCT-1 in a previous study, PCT was detected as the distance between two reflectivity spikes that appeared following the reflectivity of IOL. Peak intensity was the maximum height of the most posterior spike of PCO. The measurement was taken only at 3 points, i.e., the centre, temporal, and nasal points. Since the distribution of PCO was not even, this method may have limitations.

We further defined PCO scores by multiplying the PCO area by the mean density measured with RTVue-100 OCT, which referenced to the EPCO method [27, 28], and we proved that PCO scores were correlated with decreased visual acuity. In previous studies, the Scheimpflug photography system was used as an objective method to evaluate PCO, and the density of PCO correlated well with visual acuity [8, 13, 29]^.^ In our results, we proved that PCO scores with the Pentacam were correlated with those from RTVue-100 OCT.

When comparing pear-type and fibrosis-type PCOs, we found that the area and thickness of the pear-type cases were obviously higher than the fibrosis-type cases. However, the density of the pear-type cases was lower. The uneven proliferated and accumulated pear-type PCO led to higher scattered light. This could explain the results that pear-type PCOs affected visual acuity and contrast sensitivity to a greater extent more than the fibrosis-type PCO [30]. The higher density of the fibrosis-type PCO may be correlated with the higher Nd:YAG capsulotomy energy, as Gregor Hawlina [21] reported.

There were several limitations in this study. First, the majority of IOLs included were hydrophilic IOLs. Different IOL materials may have different light scattering properties, which may interfere with the signal intensity, although we excluded light scatter intensity from the IOL during evaluation. Thus, future studies on PCO evaluation with different IOL materials should be performed. Second, PCO could induce a decrease in visual acuity and contrast sensitivity and an increase in the scattered light; however, we only analysed the correlation between PCO severity and visual acuity. The relation between PCO severity (including area, thickness, and density), contrast sensitivity, and stray light will be the focus of a future study. Third, RTVue-100 OCT and Pentacam images were cross-sectional images of the IOL and PCO, although they were well in line with the retroillumination images and decreased visual acuity. Therefore, comparison studies between RTVue-100 OCT and other PCO evaluation methods will also be useful.

## 5. Conclusions

In summary, by using RTVue-100 OCT, we could clearly observe the IOL, posterior capsule, and the accumulated extracellular substance and LECs. The objective quantification of the PCO area, thickness, and density demonstrated a correlation between decreased visual acuity and IOL-posterior capsular space. The new objectively defined PCO score was in line with the PCO score evaluated with the Pentacam system and was correlated with the width of the IOL-posterior capsular space and the decreased visual acuity. Differences between the pear-type and fibrosis-type PCOs could also be clearly distinguished. These results suggested that RTVue-100 OCT could be used as a powerful method to evaluate the IOL-posterior capsular space and the classification and quantification of PCO.

## Figures and Tables

**Table 1:** PCO characters (area, density, and score) measured with RTVue-100 OCT.

*n*=60	Full IOL-posterior space, mean ± s.d. (range)	3 mm IOL optic region, mean ± s.d. (range)	5 mm IOL optic region, mean ± s.d. (range)
Mean area (mm^2^)	1.06 ± 0.53	0.50 ± 0.21	0.71 ± 0.38
	(0.14–2.59)	(0.05–0.88)	(0.08–1.64)
Mean density^1^	32.05 ± 14.12	46.96 ± 15.79	41.20 ± 14.00
	(6.26–99.78)	(26.39–118.44)	(22.16–113.66)
PCO score^2^	—	22.02 ± 9.27	28.07 ± 15.30
	—	(2.39–41.38)	(4.94–70.14)

^1^Averages over the area (of 3/5/6 mm diameter). ^2^PCO area × (PCO density−light scatter intensity).

**Table 2:** PCO thickness measured with RTVue-100 OCT.

*n*=60	Central point of IOL-posterior space, mean ± sd (range)	3 mm point of the IOL optic region, mean ± sd (range)	5 mm point of the IOL optic region, mean ± sd (range)
Mean thickness (mm)	0.15 ± 0.08	0.14 ± 0.07	0.12 ± 0.06
	(0.03–0.33)	(0.02–0.29)	(0.01–0.28)

## Data Availability

The data used to support the findings of this study are included within the article.
